# Tetraammine(carbonato-κ^2^ *O*,*O*′)cobalt(III) perchlorate

**DOI:** 10.1107/S1600536813018187

**Published:** 2013-07-06

**Authors:** Singaravelu Chandra Mohan, Samson Jegan Jenniefer, Packianathan Thomas Muthiah, Kandasamy Jothivenkatachalam

**Affiliations:** a Department of Chemistry, Anna University – BIT Campus, Tiruchirappalli 620 024, Tamil Nadu, India; b School of Chemistry, Bharathidasan University, Tiruchirappalli 620 024, Tamilnadu, India

## Abstract

In the title complex, [Co(CO_3_)(NH_3_)_4_]ClO_4_, both the cation and anion lie on a mirror plane. The Co^III^ ion is coordinated by two NH_3_ ligands and a chelating carbonato ligand in the equatorial sites and by two NH_3_ groups in the axial sites, forming a distorted octa­hedral geometry. In the crystal, N—H⋯O hydrogen bonds connect the anions and cations, forming a three-dimensional network.

## Related literature

For background to cobalt(III)–ammine complexes, see: Werner (1908 ▶) and to cobalt–carbonato complexes, see: McClintock *et al.* (2008 ▶); Cavigliasso *et al.* (2008 ▶). For their biological applications, see: Kumar & Thota (2005 ▶); Xu *et al.* (2009 ▶). For the chemistry of carbonato­penta­ammine­cobalt(III) and carboxyl­ato­penta­mminecobalt(III) complexes, see: Busset *et al.* (2007 ▶); Palaniappan *et al.* (2001 ▶); Jothivenkatachalam *et al.* (2013 ▶). For related Co^III^ complexes, see: Kim *et al.* (1998 ▶); Massoud *et al.* (2000 ▶); Sharma *et al.* (2004*a* ▶,*b* ▶, 2005*a* ▶,*b* ▶).
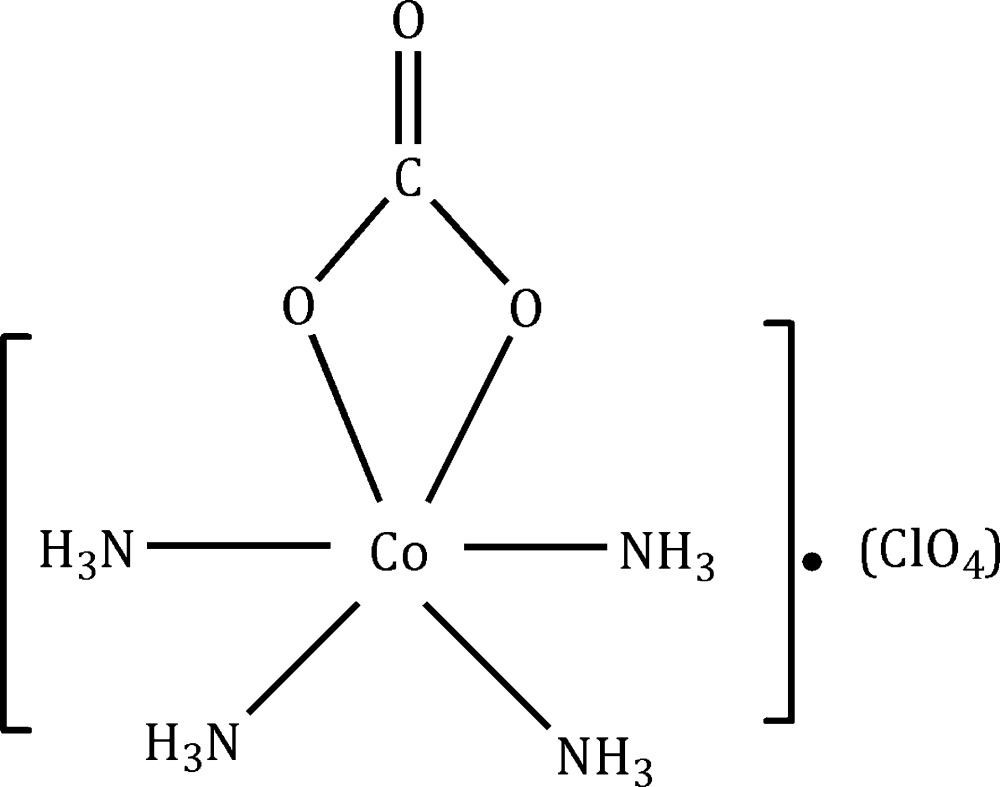


## Experimental

### 

#### Crystal data

[Co(CO_3_)(NH_3_)_4_]ClO_4_
*M* *_r_* = 286.53
Orthorhombic, 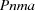

*a* = 17.8961 (5) Å
*b* = 8.0768 (2) Å
*c* = 6.8871 (2) Å
*V* = 995.48 (5) Å^3^
*Z* = 4
Mo *K*α radiation
μ = 2.01 mm^−1^
*T* = 296 K
0.09 × 0.08 × 0.07 mm

#### Data collection

Bruker SMART APEXII CCD area-detector diffractometer
Absorption correction: multi-scan (*SADABS*; Bruker, 2008 ▶) *T*_min_ = 0.951, *T*_max_ = 0.962
12900 measured reflections
1947 independent reflections
1565 reflections with *I* > 2σ(*I*)
*R*_int_ = 0.032

#### Refinement

*R*[*F* ^2^ > 2σ(*F* ^2^)] = 0.042
*wR*(*F* ^2^) = 0.153
*S* = 1.15
1947 reflections
91 parameters
H atoms treated by a mixture of independent and constrained refinement
Δρ_max_ = 1.06 e Å^−3^
Δρ_min_ = −0.75 e Å^−3^

### 

Data collection: *APEX2* (Bruker, 2008 ▶); cell refinement: *SAINT* (Bruker, 2008 ▶); data reduction: *SAINT*; program(s) used to solve structure: *SHELXS97* (Sheldrick, 2008 ▶); program(s) used to refine structure: *SHELXL97* (Sheldrick, 2008 ▶); molecular graphics: *POV-RAY* (Persistence of Vision Team, 2004 ▶) and *PLATON* (Spek, 2009 ▶); software used to prepare material for publication: *PLATON* (Spek, 2009 ▶).

## Supplementary Material

Crystal structure: contains datablock(s) global, I. DOI: 10.1107/S1600536813018187/lh5627sup1.cif

Structure factors: contains datablock(s) I. DOI: 10.1107/S1600536813018187/lh5627Isup2.hkl

Additional supplementary materials:  crystallographic information; 3D view; checkCIF report

## Figures and Tables

**Table 1:** Hydrogen-bond geometry (Å, °)

*D*—H⋯*A*	*D*—H	H⋯*A*	*D*⋯*A*	*D*—H⋯*A*
N1—H1*A*⋯O5	0.89	2.52	3.371 (6)	159
N1—H1*B*⋯O2^i^	0.89	2.18	3.017 (3)	156
N1—H1*C*⋯O3^ii^	0.89	2.58	3.063 (3)	115
N1—H1*C*⋯O2^iii^	0.89	2.58	3.313 (3)	140
N1—H1*C*⋯O1^iv^	0.89	2.59	3.311 (3)	139
N4—H2⋯O5^ii^	0.75 (3)	2.44 (3)	3.145 (4)	158 (3)
N3—H4⋯O1^iv^	0.78 (3)	2.35 (2)	3.0478 (18)	151 (3)
N3—H5⋯O2^i^	0.82 (4)	2.24 (4)	3.020 (4)	158 (3)

Symmetry codes: (i) 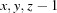; (ii) 
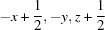
; (iii) 
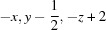
; (iv) 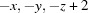.

**Fig. 1.**
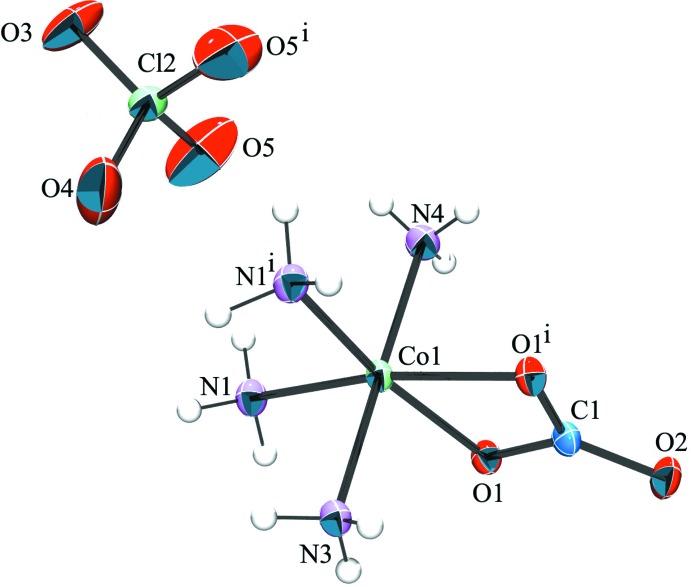
The molecular structure of the title compound, with anisotropic displacement ellipsoids drawn at 50% probability level [Symmetry code: (i) x, -y+1/2, z].

**Fig. 2.**
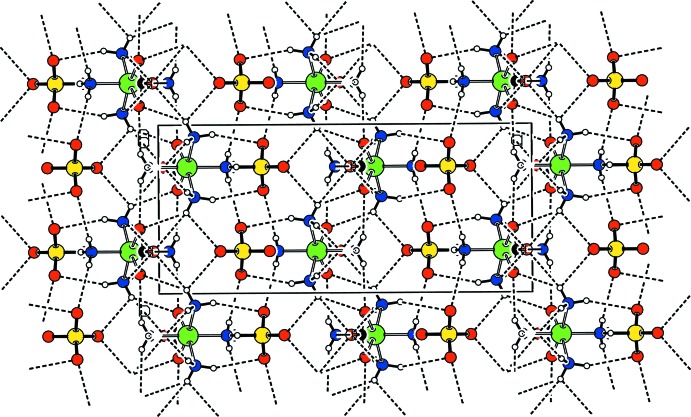
The packing of the complex viewed along the c axis, showing N—H···O hydrogen bonds as dashed lines.

## supplementary crystallographic information

### Comment

Cobalt(III) ammine complexes are well known and were widely studied by Werner (1908). In aqueous medium, the chelated ring of a bicarbonate complex is opened and protonation occurs due to hydrolysis which leads to instability. The less stability of a carbonato complex in acidic aqueous medium not only leads to protonation but also makes a site for metallation (McClintock *et al.*, 2008; Cavigliasso *et al.*, 2008). The carbanato complex also plays a vital role in photocleavage of proteins with high preference and it assists the new models of transition metal complexes for the photocleavage (Kumar & Thota, 2005). The P—O bonds present in the phosphodiester of DNA have been cleaved hydrolytically by the imitative of chelated carbonato complexes (Xu *et al.*, 2009). Recently the carbonate radical generation by photochemical reaction of carbonatopentaamminecobalt(III) complex was also reported (Busset *et al.*, 2007). The photochemical reactions of carboxylatopentamminecobalt(III) complexes lead to the reduction of metal centre and the formation of oxidized ligands, which may lead to the synthesis of value added products (Palaniappan *et al.*, 2001; Jothivenkatachalam *et al.*, 2013).

The crystal structure of the title complex is composed of one [CoCO_3_(NH_3_)_4_]^+^ cation and a ClO_4_^-^ anion in a 1:1 molar ratio. A mirror plane bisects the cation as well as the perchlorate anion, hence half a cation and an anion form the asymmetric unit. The molecular structure of the title complex is shown in Fig. 1. The Co^III^ ion is coordinated by two NH_3_ ligands and a chelating carbanato ligand equatorially, by two NH_3_ groups axially. Unlike other d^6^ octahedral Co(II) complexes the title complex shows a distortion from ideal octahedral geometry. This can be noted by the deviation of O1—Co—O1^i^ bond angle of 68.41 (7)° from the ideal octahedral bond angle of 90°. This is due to the steric restriction of the carbanato ligand in the formation of four membered chelate ring. The observed O—Co—O bond angle is similar to those observed in related [Co(CO_3_)(N)_4_]+ species (Kim *et al.*, 1998; Massoud *et al.*, 2000). The chelating CO_3_^2-^ has a slight influence on the N1—Co—N1^i^ bond angle *trans* to the O1—Co—O1^i^ angle. The N1—Co—N1^i^ bond angle is 94.22 (8)°. The Co—N bond distances observed for the complex under investigation is similar to those reported earlier [CoCO_3_(L2)]ClO_4_, [Co(NH_3_)_2_(NO_2_)_4_]^-^, [Co(NH_3_)_6_]_3_^+^, (Massoud *et al.*, 2000; Sharma *et al.*, 2004*a*,*b*; Sharma *et al.*, 2005*a*,*b*). In the crystal, N—H···O hydrogen bonds connect anions and cations to form a three-dimensional network (Fig. 2).

### Experimental

Carbonatotetramminecobalt(III) perchlorate was synthesized by the treatment of sodium bicarbonate with aquapentaamminecobalt(III) perchlorate dissolved in small amount of hot water. The pH of the reaction mixture is adjusted to pH 8 by varying sodium bicarbonate and refluxed at 333K for 4 h and kept in cool place. The purple colored carbonatotetramminecobalt(III) perchlorate precipitate then settled. The resulting solution was filtered and was dissolved in minimum amount of hot water, and then allowed to crystallize by slow evaporation at ambient temperature. Fine purple crystals of X-ray quality separated out after one week. These were filtered, washed with ethanol, acetone and air-dried.

### Refinement

The H atoms attached to N3 and N4 were located from a difference Fourier map and were refined freely. The H atoms attached to N1 were placed in geometrically idealized positions and constrained to ride on their parent atom, with N—H distance of 0.89 Å, and with *U*_iso_(H) set at 1.5U_eq_(N).

### Crystal data

**Table:**

[Co(CO_3_)(NH_3_)_4_]ClO_4_	*F*(000) = 584
*M**_r_* = 286.53	*D*_x_ = 1.912 Mg m^−^^3^
Orthorhombic, *P**n**m**a*	Mo *K*α radiation, λ = 0.71073 Å
Hall symbol: -P 2ac 2n	Cell parameters from 1947 reflections
*a* = 17.8961 (5) Å	θ = 2.3–33.0°
*b* = 8.0768 (2) Å	µ = 2.01 mm^−^^1^
*c* = 6.8871 (2) Å	*T* = 296 K
*V* = 995.48 (5) Å^3^	Plate, purple
*Z* = 4	0.09 × 0.08 × 0.07 mm

### Data collection

**Table:**

Bruker SMART APEXII CCD area-detector diffractometer	1947 independent reflections
Radiation source: fine-focus sealed tube	1565 reflections with *I* > 2σ(*I*)
Graphite monochromator	*R*_int_ = 0.032
φ and ω scans	θ_max_ = 33.0°, θ_min_ = 2.3°
Absorption correction: multi-scan (*SADABS*; Bruker, 2008)	*h* = −27→27
*T*_min_ = 0.951, *T*_max_ = 0.962	*k* = −11→12
12900 measured reflections	*l* = −10→10

### Refinement

**Table:**

Refinement on *F*^2^	Primary atom site location: structure-invariant direct methods
Least-squares matrix: full	Secondary atom site location: difference Fourier map
*R*[*F*^2^ > 2σ(*F*^2^)] = 0.042	Hydrogen site location: inferred from neighbouring sites
*wR*(*F*^2^) = 0.153	H atoms treated by a mixture of independent and constrained refinement
*S* = 1.15	*w* = 1/[σ^2^(*F*_o_^2^) + (0.1*P*)^2^] where *P* = (*F*_o_^2^ + 2*F*_c_^2^)/3
1947 reflections	(Δ/σ)_max_ < 0.001
91 parameters	Δρ_max_ = 1.06 e Å^−^^3^
0 restraints	Δρ_min_ = −0.75 e Å^−^^3^

### Special details

**Table:**

Geometry. Bond distances, angles *etc*. have been calculated using the rounded fractional coordinates. All su's are estimated from the variances of the (full) variance-covariance matrix. The cell e.s.d.'s are taken into account in the estimation of distances, angles and torsion angles
Refinement. Refinement on *F*^2^ for ALL reflections except those flagged by the user for potential systematic errors. Weighted *R*-factors *wR* and all goodnesses of fit *S* are based on *F*^2^, conventional *R*-factors *R* are based on *F*, with *F* set to zero for negative *F*^2^. The observed criterion of *F*^2^ > σ(*F*^2^) is used only for calculating -*R*-factor-obs *etc*. and is not relevant to the choice of reflections for refinement. *R*-factors based on *F*^2^ are statistically about twice as large as those based on *F*, and *R*-factors based on ALL data will be even larger.

### Fractional atomic coordinates and isotropic or equivalent isotropic displacement parameters (Å^2^)

**Table:**

	*x*	*y*	*z*	*U*_iso_*/*U*_eq_	
Co1	0.07671 (2)	0.25000	0.88852 (5)	0.0258 (1)	
O1	0.05126 (10)	0.1164 (2)	1.10933 (19)	0.0324 (5)	
O2	0.01415 (17)	0.25000	1.3801 (3)	0.0486 (9)	
N1	0.09597 (12)	0.0723 (2)	0.7015 (3)	0.0362 (5)	
N3	−0.02722 (17)	0.25000	0.8052 (4)	0.0328 (8)	
N4	0.18057 (18)	0.25000	0.9753 (5)	0.0380 (9)	
C1	0.03851 (18)	0.25000	1.2100 (4)	0.0319 (8)	
Cl2	0.27539 (5)	0.25000	0.48795 (14)	0.0426 (3)	
O3	0.3348 (3)	0.25000	0.3518 (8)	0.0950 (17)	
O4	0.2027 (3)	0.25000	0.4051 (6)	0.149 (4)	
O5	0.2799 (3)	0.1123 (4)	0.6072 (7)	0.131 (2)	
H1A	0.14500	0.05620	0.69100	0.0540*	
H1B	0.07740	0.10050	0.58620	0.0540*	
H1C	0.07430	−0.02060	0.74220	0.0540*	
H2	0.1869 (18)	0.174 (4)	1.035 (5)	0.052 (10)*	
H3	0.209 (3)	0.25000	0.904 (8)	0.062 (19)*	
H4	−0.0472 (17)	0.171 (3)	0.843 (4)	0.032 (7)*	
H5	−0.029 (2)	0.25000	0.686 (6)	0.023 (8)*	

### Atomic displacement parameters (Å^2^)

**Table:**

	*U*^11^	*U*^22^	*U*^33^	*U*^12^	*U*^13^	*U*^23^
Co1	0.0320 (3)	0.0241 (2)	0.0213 (2)	0.0000	−0.0021 (1)	0.0000
O1	0.0417 (9)	0.0285 (8)	0.0269 (7)	−0.0032 (7)	−0.0017 (5)	0.0027 (5)
O2	0.0462 (14)	0.077 (2)	0.0227 (10)	0.0000	0.0028 (8)	0.0000
N1	0.0450 (10)	0.0319 (9)	0.0318 (8)	0.0014 (8)	0.0007 (8)	−0.0035 (7)
N3	0.0368 (13)	0.0343 (14)	0.0272 (12)	0.0000	−0.0029 (10)	0.0000
N4	0.0369 (14)	0.0407 (18)	0.0364 (14)	0.0000	−0.0037 (12)	0.0000
C1	0.0336 (13)	0.0392 (16)	0.0230 (11)	0.0000	−0.0044 (10)	0.0000
Cl2	0.0418 (4)	0.0377 (5)	0.0482 (5)	0.0000	0.0064 (3)	0.0000
O3	0.102 (3)	0.068 (3)	0.115 (3)	0.0000	0.076 (3)	0.0000
O4	0.066 (3)	0.324 (11)	0.056 (3)	0.0000	−0.0011 (19)	0.0000
O5	0.162 (4)	0.0684 (19)	0.161 (4)	0.041 (2)	0.070 (3)	0.054 (2)

### Geometric parameters (Å, º)

**Table:**

Co1—O1	1.9195 (15)	O2—C1	1.250 (4)
Co1—N1	1.9590 (19)	N1—H1A	0.8900
Co1—N3	1.947 (3)	N1—H1B	0.8900
Co1—N4	1.952 (3)	N1—H1C	0.8900
Co1—O1^i^	1.9195 (15)	N3—H4	0.78 (3)
Co1—N1^i^	1.9590 (19)	N3—H5	0.82 (4)
Cl2—O5^i^	1.385 (4)	N3—H4^i^	0.78 (3)
Cl2—O3	1.418 (6)	N4—H3	0.71 (5)
Cl2—O4	1.421 (5)	N4—H2^i^	0.75 (3)
Cl2—O5	1.385 (4)	N4—H2	0.75 (3)
O1—C1	1.303 (2)		
			
Co1···O2^ii^	3.676 (2)	N3···O1	2.743 (3)
Co1···O1^iii^	3.7420 (17)	N3···N1^i^	2.726 (3)
Co1···O1^iv^	3.7420 (17)	N3···O2^ii^	3.020 (4)
Co1···O2^v^	3.676 (2)	N3···N1	2.726 (3)
Cl2···H1A^i^	3.1400	N3···C1	3.026 (4)
Cl2···H1A	3.1400	N4···N1^i^	2.812 (4)
Cl2···H3	3.10 (5)	N4···O1	2.715 (3)
Cl2···H3	3.10 (5)	N4···O4^vii^	2.987 (5)
O1···O2	2.255 (2)	N4···O1^i^	2.715 (3)
O1···N3^iv^	3.0478 (18)	N4···O5^xiv^	3.145 (4)
O1···N3	2.743 (3)	N4···N1	2.812 (4)
O1···Co1^vi^	3.7420 (17)	N4···C1	3.013 (5)
O1···N1	2.942 (2)	N4···O4^viii^	2.987 (5)
O1···N4	2.715 (3)	N4···O5^xii^	3.145 (4)
O1···O1^iv^	3.028 (2)	N1···H5	2.66 (3)
O1···N3^vi^	3.0478 (18)	N1···H3	2.85 (5)
O1···O1^i^	2.158 (2)	N1···H4	2.86 (3)
O1···Co1^iv^	3.7420 (17)	N1···H1B^i^	2.7800
O2···N1^vii^	3.017 (3)	N1···H2	2.93 (3)
O2···N3^vii^	3.020 (4)	N3···H1C^i^	2.8800
O2···Co1^viii^	3.676 (2)	N3···H1B	2.6900
O2···O1^i^	2.255 (2)	N3···H1C	2.8800
O2···N3^viii^	3.020 (4)	N3···H1B^i^	2.6900
O2···Co1^vii^	3.676 (2)	N4···H1A^i^	2.5900
O2···O1	2.255 (2)	N4···H1A	2.5900
O2···N1^viii^	3.017 (3)	C1···O4^viii^	3.231 (6)
O3···N1^ix^	3.063 (3)	C1···N3	3.026 (4)
O3···N1^x^	3.063 (3)	C1···N4	3.013 (5)
O4···N1^i^	3.143 (5)	C1···O4^vii^	3.231 (6)
O4···N4^ii^	2.987 (5)	C1···H2	2.98 (3)
O4···C1^v^	3.231 (6)	C1···H1C^iv^	2.7600
O4···C1^ii^	3.231 (6)	C1···H4	3.03 (3)
O4···N4^v^	2.987 (5)	C1···H1B^viii^	2.9400
O4···N1	3.143 (5)	C1···H4^i^	3.03 (3)
O5···N4^xi^	3.145 (4)	C1···H1C^iii^	2.7600
O5···N4^ix^	3.145 (4)	C1···H1B^vii^	2.9400
O1···H1C^iv^	2.5900	C1···H2^i^	2.98 (3)
O1···H2	2.52 (3)	H1A···H3	2.4300
O1···H4^iv^	2.35 (2)	H1A···O3^xii^	2.7300
O1···H4	2.58 (3)	H1A···O3^xiii^	2.7300
O1···H1C	2.7900	H1A···O5	2.5200
O2···H1C^iv^	2.5800	H1A···Cl2	3.1400
O2···H5^viii^	2.24 (4)	H1A···O4	2.7200
O2···H1B^vii^	2.1800	H1A···Cl2	3.1400
O2···H1C^iii^	2.5800	H1A···O4	2.7200
O2···H5^vii^	2.24 (4)	H1B···O2^v^	2.1800
O2···H1B^viii^	2.1800	H1B···C1^ii^	2.9400
O3···H1A^x^	2.7300	H1B···H5	2.3600
O3···H1A^ix^	2.7300	H1B···O2^ii^	2.1800
O3···H1C^ix^	2.5800	H1B···O4	2.8400
O3···H1C^x^	2.5800	H1B···O4	2.8400
O4···H1A	2.7200	H1B···C1^v^	2.9400
O4···H2^ii^	2.64 (3)	H1B···H1B^i^	2.4100
O4···H1B	2.8400	H1C···C1^vi^	2.7600
O4···H2^v^	2.64 (3)	H1C···O1^iv^	2.5900
O4···H1A^i^	2.7200	H1C···O2^iv^	2.5800
O4···H1B^i^	2.8400	H1C···O3^xii^	2.5800
O5···H3	2.65 (5)	H1C···O2^vi^	2.5800
O5···H1A	2.5200	H1C···C1^iv^	2.7600
O5···H2^ix^	2.44 (3)	H1C···O3^xiii^	2.5800
O5···H3	2.65 (5)	H2···O4^viii^	2.64 (3)
N1···O1	2.942 (2)	H2···O4^vii^	2.64 (3)
N1···O4	3.143 (5)	H2···O5^xii^	2.44 (3)
N1···N1^i^	2.871 (2)	H3···Cl2	3.10 (5)
N1···N3	2.726 (3)	H3···O5	2.65 (5)
N1···O2^ii^	3.017 (3)	H3···H1A	2.4300
N1···O4	3.143 (5)	H3···Cl2	3.10 (5)
N1···O3^xii^	3.063 (3)	H3···O5^i^	2.65 (5)
N1···N4	2.812 (4)	H3···H1A^i^	2.4300
N1···O2^v^	3.017 (3)	H4···O1^iv^	2.35 (2)
N1···O3^xiii^	3.063 (3)	H5···O2^v^	2.24 (4)
N3···O2^v^	3.020 (4)	H5···H1B^i^	2.3600
N3···O1^iv^	3.0478 (18)	H5···H1B	2.3600
N3···O1^i^	2.743 (3)	H5···O2^ii^	2.24 (4)
N3···O1^iii^	3.0478 (18)		
			
O1—Co1—N1	98.67 (7)	Co1—O1—C1	89.86 (13)
O1—Co1—N3	90.39 (9)	Co1—N1—H1C	110.00
O1—Co1—N4	89.05 (10)	Co1—N1—H1B	109.00
O1—Co1—C1	34.21 (5)	H1A—N1—H1C	109.00
O1—Co1—O1^i^	68.41 (7)	H1B—N1—H1C	110.00
O1—Co1—N1^i^	167.04 (7)	H1A—N1—H1B	109.00
N1—Co1—N3	88.53 (8)	Co1—N1—H1A	110.00
N1—Co1—N4	91.94 (9)	H4—N3—H4^i^	111 (3)
N1—Co1—C1	132.85 (6)	H4^i^—N3—H5	109 (2)
O1^i^—Co1—N1	167.04 (7)	H4—N3—H5	109 (2)
N1—Co1—N1^i^	94.22 (8)	Co1—N3—H4^i^	110 (2)
N3—Co1—N4	179.32 (13)	Co1—N3—H4	110 (2)
N3—Co1—C1	89.99 (11)	Co1—N3—H5	109 (3)
O1^i^—Co1—N3	90.39 (9)	H2^i^—N4—H3	106 (3)
N1^i^—Co1—N3	88.53 (8)	Co1—N4—H2^i^	108 (2)
N4—Co1—C1	89.33 (13)	Co1—N4—H2	108 (2)
O1^i^—Co1—N4	89.05 (10)	Co1—N4—H3	118 (4)
N1^i^—Co1—N4	91.94 (9)	H2—N4—H2^i^	110 (4)
O1^i^—Co1—C1	34.21 (5)	H2—N4—H3	106 (3)
N1^i^—Co1—C1	132.85 (6)	Co1—C1—O2	176.8 (3)
O1^i^—Co1—N1^i^	98.67 (7)	Co1—C1—O1^i^	55.93 (12)
O5—Cl2—O5^i^	106.9 (3)	O1—C1—O1^i^	111.9 (2)
O3—Cl2—O5	110.4 (2)	O1^i^—C1—O2	124.05 (12)
O3—Cl2—O5^i^	110.4 (2)	O1—C1—O2	124.05 (12)
O3—Cl2—O4	114.9 (3)	Co1—C1—O1	55.93 (12)
O4—Cl2—O5	107.0 (2)		
			
N1—Co1—O1—C1	−177.97 (17)	N3—Co1—C1—O1	90.72 (15)
N3—Co1—O1—C1	−89.40 (17)	N4—Co1—C1—O1	−89.29 (15)
N4—Co1—O1—C1	90.22 (17)	O1^i^—Co1—C1—O1	−178.6 (3)
O1^i^—Co1—O1—C1	0.87 (16)	N1^i^—Co1—C1—O1	178.69 (14)
O1—Co1—C1—O1^i^	178.6 (3)	O1—Co1—O1^i^—C1	−0.87 (16)
N1—Co1—C1—O1	2.7 (2)	Co1—O1—C1—O2	176.1 (3)
N1—Co1—C1—O1^i^	−178.69 (14)	Co1—O1—C1—O1^i^	−1.3 (2)

Symmetry codes: (i) *x*, −*y*+1/2, *z*; (ii) *x*, *y*, *z*−1; (iii) −*x*, *y*+1/2, −*z*+2; (iv) −*x*, −*y*, −*z*+2; (v) *x*, −*y*+1/2, *z*−1; (vi) −*x*, *y*−1/2, −*z*+2; (vii) *x*, −*y*+1/2, *z*+1; (viii) *x*, *y*, *z*+1; (ix) −*x*+1/2, −*y*, *z*−1/2; (x) −*x*+1/2, *y*+1/2, *z*−1/2; (xi) −*x*+1/2, *y*−1/2, *z*−1/2; (xii) −*x*+1/2, −*y*, *z*+1/2; (xiii) −*x*+1/2, *y*−1/2, *z*+1/2; (xiv) −*x*+1/2, *y*+1/2, *z*+1/2.

### Hydrogen-bond geometry (Å, º)

**Table:**

*D*—H···*A*	*D*—H	H···*A*	*D*···*A*	*D*—H···*A*
N1—H1*A*···O5	0.89	2.52	3.371 (6)	159
N1—H1*B*···O2^ii^	0.89	2.18	3.017 (3)	156
N1—H1*C*···O3^xii^	0.89	2.58	3.063 (3)	115
N1—H1*C*···O2^vi^	0.89	2.58	3.313 (3)	140
N1—H1*C*···O1^iv^	0.89	2.59	3.311 (3)	139
N4—H2···O5^xii^	0.75 (3)	2.44 (3)	3.145 (4)	158 (3)
N3—H4···O1^iv^	0.78 (3)	2.35 (2)	3.0478 (18)	151 (3)
N3—H5···O2^ii^	0.82 (4)	2.24 (4)	3.020 (4)	158 (3)

Symmetry codes: (ii) *x*, *y*, *z*−1; (iv) −*x*, −*y*, −*z*+2; (vi) −*x*, *y*−1/2, −*z*+2; (xii) −*x*+1/2, −*y*, *z*+1/2.
